# Hypertension and Diabetes Cooperatively Drive HSP90 Activation, HSP70 Suppression, and Left Ventricular Interstitial Expansion: Relevance to Maladaptive Myocardial Remodeling

**DOI:** 10.3390/pathophysiology33010019

**Published:** 2026-02-18

**Authors:** Anastasia P. Sklifasovskaya, Mikhail L. Blagonravov, Madina M. Azova, Sergey V. Kurevlev, Vyacheslav A. Goryachev, Sergey P. Syatkin, Tatyana Yu. Zotova, Daniil Yu. Prokofiev

**Affiliations:** 1 Institute of Medicine, RUDN University, 6 Miklukho-Maklaya St., Moscow 117198, Russia; azova-mm@rudn.ru (M.M.A.); kurevlev-sv@rudn.ru (S.V.K.); goryachev-va@rudn.ru (V.A.G.); syatkin-sp@rudn.ru (S.P.S.); zotova-tyu@rudn.ru (T.Y.Z.); prokofyev-dyu@rudn.ru (D.Y.P.); 2 Moscow Botkin Multidisciplinary Clinical and Scientific Center, Moscow 125284, Russia

**Keywords:** myocardial damage, HSP90, HSP70, insulin dependent diabetes mellitus, arterial hypertension

## Abstract

Background: Arterial hypertension (AH) and insulin-dependent diabetes mellitus (DM) are major comorbid risk factors for accelerated myocardial damage, yet the behavior of key stress-adaptive heat shock proteins HSP70 and HSP90 under combined stress remains unclear. This study aimed to characterize the expression profiles of HSP70 and HSP90 in left ventricular cardiomyocytes during isolated and comorbid AH and DM, and to evaluate their association with structural remodeling and expansion of interstitial elements. Methods: The study was conducted in accordance with the European Convention for the Protection of Vertebrate Animals (ethical approval No. 26, RUDN Institute of Medicine, 18 February 2021) on 25 male rats divided into five groups (*n* = 5 each): control—38-week-old Wistar–Kyoto (WKY) rats; AH—38-week-old spontaneously hypertensive rats (SHR); long-term AH—57-week-old SHR; DM—38-week-old WKY rats with streptozotocin-induced insulin-dependent DM (65 mg/kg, i.p.); AH+DM—38-week-old SHR with STZ-induced DM. After 30 days of DM, left ventricular (LV) tissue was analyzed by immunohistochemistry (IHC) for HSP70/HSP90 protein expression and by RT-qPCR for mRNA levels. Increased stromal elements in myocardium were quantified morphometrically as interstitial stromal volume fraction (%) on hematoxylin and eosin-stained sections. Results: HSP90 was significantly upregulated in all pathological groups. The most pronounced increase occurred in isolated DM, with a 4.0-fold rise in HSP90-positive area (21.80% vs. 5.45% in control) and a 1.82-fold increase in mRNA. In the AH+DM group, HSP90 mRNA expression was extremely elevated (25.93-fold), accompanied by a 3.7-fold increase in protein. In contrast, HSP70 protein was elevated only in the 38-week AH group (27.68% vs. 19.70% control, *p* ≤ 0.05), remained unchanged in isolated DM (19.50%), and was significantly reduced in AH+DM (14.71%, *p* ≤ 0.05), despite a modest 1.64-fold mRNA upregulation in DM. Morphometric analysis revealed progressive expansion of interstitial elements, most severe in AH+DM (9.43% stromal volume vs. 4.81% in control, *p* ≤ 0.05). Conclusions: Comorbid AH and DM provoke synergistic HSP90 upregulation, while HSP70 expression is markedly suppressed, indicating a shift from an adaptive to a maladaptive cellular-stress response. The imbalance between HSP90 and HSP70 may represent a key molecular mechanism underlying accelerated structural and functional deterioration of the myocardium in cardiometabolic comorbidity.

## 1. Introduction

Constant monitoring of protein quality in cells is essential to ensure the contractile and metabolic requirements of the heart, both under normal and pathological conditions. Molecular chaperones play a crucial role in repairing proteins, mainly in the sarcoplasmic reticulum, sarcomeres, and mitochondria, which helps to minimize stress on the heart muscle and prevents heart failure [1]. Cardiovascular diseases are often characterized by increased mechanical and oxidative stress, as well as changes in pH levels in cardiomyocyte (CMC) cytoplasm. These changes may lead to the accumulation of misfolded proteins, which can be toxic to CMCs and directly cause heart failure [2] or disrupt metabolism, inducing premature apoptosis or autophagy [3]. One of the most significant classes of molecular chaperones, which help regulate the balance between protein synthesis and degradation, assist in refolding incorrectly folded proteins, and can protect CMCs from death, are heat shock proteins [4].

Heat shock proteins 70 and 90 (HSP70 and HSP90), along with their co-chaperones CHIP and BAG-3, play a crucial role in maintaining the integrity of CMCs during the action of various stressors. These proteins are the first line of defense for CMCs, as they protect these cells from damage. When proteins are not properly folded, HSP90 helps them stay bound to it until they can be re-folded. If this does not happen, the protein remains bound to HSP90, which prevents it from aggregating with other misfolded proteins. HSP90 also transfers these proteins to the HSP70 complex, where they can be unfolded and refolded again [5,6,7]. The production of heat shock proteins in CMCs in response to stress is important for a number of processes, such as sarcomere assembly, autophagy, and normal protein metabolism. The UPR (unfolded protein response) system also plays a role in these processes [8].

The HSP70 family of molecular chaperones is composed of monomeric proteins that are found throughout the cytosol, cell membranes, and extracellular space [9]. Under normal conditions, these chaperones ensure the proper folding of newly synthesized proteins and the maintenance of homeostasis in the intracellular environment [5]. They also play an important role in chaperone-mediated autophagy, a process that is responsible for the destruction of damaged or abnormal proteins. The heat shock cognate protein 70 (HSC70 complex), which is a member of the HSP70 family, recognizes and binds to proteins that contain the KFERQ motif. These proteins can be oxidized or abnormally folded. The HSC70 then delivers these proteins to the lysosome, where they are broken down. The HSC70 also binds to a receptor on the lysosome-bound membrane, known as LAMP2A, to initiate this process [10]. Recent research has shown that the HSP70 family is not only responsible for protein degradation, but it is also involved in the assembly of the proteasome [11], another important cellular component that helps break down proteins. This suggests that these chaperones play a crucial role in maintaining the health and function of cells.

A pathological process in the heart muscle (for example, hypertrophy or ischemia) can lead to an increase in the number of misfolded proteins that need to be refolded into their natural state or removed to prevent protein aggregation [12]. Transgenic mice overexpressing HSP70 showed higher peak systolic pressure and less cell damage after ischemia–reperfusion [13]. In addition, there appears to be a direct correlation between the level of HSP70 expression and the degree of myocardial protection [14]. This observation was confirmed by the finding that adenovirus-mediated HSP70 gene transfer reduced the size of the infarct area by almost 50% in rabbits compared to those injected with saline solution. Together, these results suggest that increased expression of molecular chaperones, such as HSP70, during myocardial ischemia plays a protective role in the heart [15].

HSP90 is a major stress-adaptive protein that is responsible for the proper folding, activation, transport, stabilization, and degradation of other proteins in the cell. It is composed of cytosolic HSP90a, HSP90b, TRAP1, and Grp94 isoforms that are localized in the mitochondria and endoplasmic reticulum (ER). The stress-adaptive effects of HSP90 include the inhibition of the expression of components of the complement system, cytokines, and JNK, which play an important role in inflammation and apoptosis after ischemia/reperfusion (I/R) injury. This helps to reduce myocardial damage and apoptosis. It was found that HSP90 plays a crucial role in ischemic postconditioning, a phenomenon that occurs after I/R injury and is responsible for cardiac protection. It was shown that HSP90 inhibited the expression of C3, C5a, TNFα, IL-1β, and JNK [16].

Damage to microvessels of the heart is a common complication of diabetes, which occurs as a result of increased inflammation and the induction of a UPR. While UPR is an adaptive mechanism, overactivation of this response triggers a cascade of events leading to cell death in tissues and organs. The inositol-dependent enzyme 1 alpha (IRE1α) plays a critical role in this process, contributing to the production of X-box binding protein 1 (XBP1). This protein then speeds up the degradation of vascular endothelial growth factor A (VEGF A), which is essential for the repair of damaged blood vessels [17]. The stability of IRE1α is determined by its association with the chaperone protein HSP90 [18]. When UPR is activated, the IRE1α-HSP90 complex is formed, leading to endothelial dysfunction and further damage to microvessels of the heart. In addition, diabetes-induced hyperglycemia leads to the glycation of proteins and lipids, known as advanced glycation end products (AGEs), which have been shown to cause UPR and ER stress, leading to apoptosis and disruption of endothelial integrity and function [19]. Furthermore, the protective role of CMCs against damage caused by high glucose levels has been demonstrated for both HSP90 via the Akt pathway and TRAP1 through regulation of mitochondrial membrane potential (MMP) and opening of the mitochondrial permeability transition pore (MPTP), as well as regulation of ROS levels [20].

This study is a continuation of a series of studies devoted to the role of heat shock proteins in the pathogenesis of myocardial alteration caused by a combination of arterial hypertension and diabetes mellitus [21,22,23]. The combination of hypertension with diabetes increases the risk of adverse cardiovascular events even in the case of masked hypertension compared with clinical situations in the absence of such a combination, which was shown in the HONEST study [24].

In our previous studies [21,22], we used the same models and studied the low-molecular ATP-independent heat shock proteins HSP10, HSP27, and mitochondrial HSP60. As a rule, chaperones are paired; for example, HSP60, localized mainly in the mitochondria, has HSP10 as a co-chaperone, and HSP70 protein, HSP27 is a co-chaperone. At the same time, these co-chaperones can be expressed in different ways depending on the type of alteration. Thus, the basal ratio of HSP60/HSP10 proteins is 2/1, and this ratio changes with alterations of various origins, which indicates that the operation of this chaperone machine is inhibited [23].

Also, in a previous study on the same models, we evaluated the expression of antioxidants TXNIP and GS in order to assess the metabolism in the myocardium and the functional state of antioxidant systems in general, as this also affects the activity of heat shock proteins [25].

This study will allow us to supplement information about the processes already studied [26,27,28,29] and describe more specifically the pathogenetic pathways that are activated during these types of myocardial alterations in the future.

Despite the availability of a significant amount of data on the involvement of heat shock proteins HSP90 and HSP70 in cellular responses to the action of pathological factors in various diseases, there is still no clear understanding of their role in myocardial damage caused by arterial hypertension (AH) and diabetes mellitus (DM).

The aim of our study was to investigate the expression patterns of ATP-dependent heat shock proteins HSP90 and HSP70 in LV cardiomyocytes in AH, insulin-dependent DM, and in a combination of these conditions.

## 2. Materials and Methods

### 2.1. Animals and Housing

The experiment was carried out on 25 male rats, including 10 Wistar–Kyoto (WKY) and 15 SHR (spontaneously hypertensive) rats. The animals were obtained from the Nursery for Laboratory Animals “Pushchino” (branch of the Shemyakin–Ovchinnikov Institute of Bioorganic Chemistry of the Russian Academy of Sciences). Prior to the start of the investigation, all the animals were acclimatized for 2 weeks in the laboratory where the experiment was carried out. During the experiment, each animal was kept in an individual cage with artificial light under a free-motion regimen and with free access to water and food. The animals were consistently fed at the same time, 19:00 h. The room was kept at a constant temperature of +23 °C. The rats with DM received a diet adapted for diabetic animals. The blood pressure of SHRs was between 180 and 200 mm of mercury for the systolic pressure and between 140 and 150 mm for the diastolic pressure at the time they were selected for the experimental groups. The experiment was carried out in accordance with the European Convention for the Protection of Vertebrate Animals used for Experimental and Other Scientific Purposes (Strasbourg, 18.III.1986) and was also approved by the Ethical Committee of the RUDN Institute of Medicine, an ethical code number 26, date: 18 February 2021.

### 2.2. Experimental Design

The study involved 10 WKY and 15 SHRs weighing between 290 and 400 g. The animals were divided into five groups: group 1 (control group)—intact normotensive Wistar–Kyoto rats aged 38 weeks; group 2—hypertensive SHRs aged 38 weeks; group 3—hypertensive SHRs aged 57 weeks; group 4—normotensive Wistar–Kyoto rats with 30 days insulin-depended diabetes mellitus aged 38 weeks; group 5—hypertensive SHRs with 30 day insulin-depended diabetes mellitus aged 38 weeks. Each group consisted of 5 rats.

Wistar–Kyoto (WKY) rats are a well-established normotensive control strain that do not develop spontaneous hypertension at any age, unlike their genetic counterpart, SHRs. The age of 38 weeks was selected to ensure maturity (equivalent to ~9 human years) and to minimize the influence of acute compensatory mechanisms that dominate in younger animals. This age also allows direct comparison with age-matched SHRs, in which stable, non-compensated cardiac alterations are reliably observed by this stage [21].

Groups 4 and 5 received a single intraperitoneal injection of streptozotocin (STZ, 65 mg/kg) to induce insulin-deficient diabetes. Thus, group 5 represents comorbid animals: 38-week-old SHRs with both genetic arterial hypertension and STZ-induced diabetes.

### 2.3. Modeling of Insulin-Dependent DM

Modeling of insulin-dependent DM in groups 4 and 5 of animals was carried out using a single intraperitoneal injection of Streptozotocin (Alfa Aesar, Ward Hill, MA, USA) at a dose of 65 mg/kg body weight. The Streptozotocin solution was prepared immediately before injection on a citrate buffer and administered at a temperature of +4 °C. After 3 days, glucose levels were measured using an AccuChek Active glucose meter (Roche Diabetes Care GmbH, Mannheim, Germany) in the blood taken from the tail veins of the animals. Animals with glucose levels above 16 mmol/L were selected for further experimentation. Following confirmation of diabetes (blood glucose >16 mmol/L on day 3), all STZ-injected rats developed sustained hyperglycemia. Throughout the 30-day experimental period, non-fasted blood glucose levels in diabetic animals (both WKY and SHR) ranged between 25.5 and 33.3 mmol/L, as measured daily using an AccuChek Active glucometer (Roche Diabetes Care GmbH, Germany). No insulin therapy was administered. Measurements were taken on a daily basis. In the present study, we reused specimens of such groups.

### 2.4. Method for Assessing the Levels of Ketonuria and Glucosuria in the Urine of SHRs with Streptozotocin-Induced Diabetes

In SHRs with insulin-dependent diabetes induced by streptozotocin, the levels of ketone bodies and glucose were evaluated in urine samples collected on day 25 after induction of diabetes. For this purpose, the animals were kept in a chamber for 4–5 h each day to collect a daily urine sample. Subsequently, a Ketogluc-1 test strip (manufactured by Biosensor AN LLC, Nizhny Novgorod, Russia) was used to qualitatively and semi-quantitatively determine the presence of glucose and ketone bodies in the urine, according to the manufacturer’s instructions. After a 4–5 s incubation period, the strip was removed, and excess fluid was gently removed from the sensor elements by touching the edge of the strip against clean filter paper for 2–3 s. The indicator strip was then placed on a flat, dry, clean surface with the sensor facing upward. After 2 min of the sensor elements being immersed in urine, the color of each element was compared to the corresponding color scale on the kit packaging in good lighting conditions. A change in color indicated the presence of ketones and glucose in the sample (qualitative analysis). Semi-quantitative analysis was performed by comparing the colors of the elements to the corresponding colors on the scale. The detectable range for ketones in urine was 0.0–16.0 mmol/L, with a minimum detectable concentration of 0.5 mmol/L. Glucose concentrations were detected in the range 0.0–2.0%, corresponding to 0.0–112 mmol/L. The color scale on the label has 6 color bands corresponding to glucose levels in % (mmol/L): 0.0 (0.0), 0.1 (5.6), 0.25 (14.0), 0.5 (28.0), 1.0 (56.0), and 2.0 (112). The lowest detectable glucose level in urine is 0.1% (5.6 mmol/L) or less.

### 2.5. Method for Assessment of Some Functional Parameters of the Cardiovascular System

Parameters of the functional activity of the cardiovascular system were studied using a telemetric monitoring technique using the radio telemetry system DSi (Data Sciences International, St. Paul, MN, USA). For this purpose, DSi HD-S11 radio transmitters were surgically implanted, under general anesthesia, into the animals. BP was monitored using a catheter installed in the lumen of the abdominal aorta and fixed with a tissue hemostatic adhesive. The obtained data were processed using the DataquestA.R.T.4.2 Gold (Data Sciences International, St. Paul, MN, USA) and ChronosFit 1.30 (Emka Technologies, Paris, France) software. The following parameters were determined: 24 h middle systolic blood pressure (BPsyst), 24 h middle diastolic blood pressure (BPdiast), and 24 h middle heart rate (HR).

### 2.6. Morphological and Immunohistochemical Study

In the animals of the above-mentioned groups, thoracotomy and cardiac excision were performed under general anesthesia, resulting in humane euthanasia according to the approved ethical protocol. The collected left ventricular (LV) myocardial samples were fixed in 10% neutral buffered formaldehyde for 72 h. The material was then processed and embedded in paraffin according to the generally accepted method. Histological sections with a thickness of 5 μm were made using the microtome “Slidt 2003” (SLEE Medical GmbH, Mainz, Germany) and placed on slides with a poly-L-lysine coating (for immunohistochemical analysis) or on ordinary slides (for histological examination).

Sections for immunohistochemical examination were dewaxed using xylene and then processed with alcohols of decreasing concentration. To assess the expression of HSP70 and HSP90 in CMCs, primary antibodies against HSP70 and HSP90 produced in rabbits (Sigma-Aldrich, St. Louis, MO, USA) were used. The results of the immunohistochemistry reaction were visualized with the use of a set of reagents, “Rabbit-specific HRP/DAB (ABC) Detection IHC Kit” (Abcam, Cambridge, UK) Preparations were also stained with Mayer’s hematoxylin. A reaction was considered to be positive if brown staining appeared in the cytoplasm of CMCs. Both HSP70 and HSP90 exhibited exclusively cytoplasmic immunoreactivity in cardiomyocytes, with diffuse granular DAB staining distributed throughout the sarcoplasm and consistently absent from nuclei. The signal aligned with the cross-striated morphology of cardiac myofibrils, confirming intracellular localization within contractile cytoplasm. No specific sub-organellar (e.g., mitochondrial or sarcoplasmic reticular) or membrane-associated patterns were resolved under light microscopy (×400). In diabetic conditions (DM, AH+DM), both chaperones showed additional cytoplasmic staining in perivascular smooth muscle cells, whereas endothelial cells remained negative. Light microscopy was performed on 30 randomly selected fields in each myocardial section at a magnification of 400× using a Nikon Eclipse E-400 microscope (Nikon Corporation, Tokyo, Japan) equipped with the Watec 221S video camera. Quantitative analysis of positively stained cardiac muscle cells was performed. Quantification was based on the proportion of cytoplasmic area showing specific brown DAB staining, a validated stereological approach for comparative IHC analysis. The ratio of the number of evenly spaced points within the positively stained cytoplasm of cardiac muscle cells to the total number of cytoplasmic points was determined. For uniformity, only transmural mid-wall sections (avoiding subepicardial and subendocardial regions) were analyzed.

For consistent spatial sampling, all immunohistochemical and morphometric analyses were performed exclusively on mid-wall transmural sections of the left ventricle, avoiding subepicardial and subendocardial zones. This approach ensured comparability across all experimental groups.

All quantitative immunohistochemical analyses were performed exclusively on cardiomyocyte cytoplasm in the mid-myocardial layer, excluding vascular and interstitial cells. Vascular staining was assessed qualitatively but not included in point-counting but assessed qualitatively. Scale bars (50 μm) have been added to all micrographs for spatial reference.

### 2.7. RT-PCR Analysis of HSP70 and HSP90 mRNA Content

In animals from the above groups, thoracic incisions and cardiac excisions were performed under general anesthesia. Intact RNA (Eurogen, Moscow, Russia) was added to the collected left ventricular myocardial tissue samples for RNA stabilization and long-term preservation at −80 °C. Thereafter, RNA was extracted from cells using the ExtractRNA kit (Eurogen, Moscow, Russia), and reverse transcription was carried out using the MMLV RT kit (Eurogen, Moscow, Russia). Expression of HSP70 and HSP90 mRNA was quantified using a mixture of qPCR mix-HS SYBR (Eurogen, Moscow, Russia) and primers synthesized by Eurogen, using PCR-RV, relative to the expression level of *GAPDH* on an amplifier CFX96 instrument (Bio-Rad Laboratories, Hercules, CA, USA), based on TaqMan technology. The relative abundance of mRNA of the HSP60 gene was calculated by directly comparing data using the following formula: [A]_0_/[B]_0_ = E^ΔC(T)^, where [A]_0_ represents the initial concentration of gene mRNA in the PCR reaction mixture, and [B]_0_ represents the initial concentration of GAPDH mRNA in the reaction mixture. E is the effectiveness of the reaction (assumed to be 1.98). ΔC(T) represents the difference between the threshold cycles for *GAPDH* and the target gene. Oligonucleotide sequences used for PCR-PB are as follows: *GAPDH*: F = TGACAACTCCCTCAAGATTGTC; R = GGCATGGACTGTGGTCATGA; *HSP70*: F = TCAGACTGCTATGTCGCTG; R = GAAAGAAACACAAGCCGGCG; *HSP90*: F = TACTATACAGAGGGCGGGGG; R = AAACTAGGCCTGGGCATTGA.

### 2.8. Statistics

Statistical data analysis was performed using Statistica 6.0 software (StatSoft Inc., Tulsa, OK, USA). For each parameter, the mean value and standard error were calculated. To determine the significance of differences between groups, the Mann–Whitney U test was used (the difference between the mean values was regarded as significant at *p* ≤ 0.05). To account for multiple testing, *p*-values were adjusted using the Holm–Bonferroni method. All reported significant differences remained significant after correction (adjusted *p* ≤ 0.05). Given our hypothesis-driven design (comparison of each group with control) and small sample size (*n* = 5), post hoc correction for multiple comparisons was not applied, in accordance with recommendations for preclinical studies [30].

## 3. Results

### 3.1. Ketonuria and Glucosuria Levels in SHRs with Streptozotocin-Induced Diabetes

The level of ketone bodies in urine was evaluated to determine the development of ketoacidosis in the rats. When assessing this indicator, all signs of ketosis were observed, including high blood glucose levels (>20 mmol/L) following a low-carbohydrate diet, increased thirst (with one animal consuming approximately 250–300 mL of water per day), weight loss, general lethargy, and decreased activity.

For example, we present the results of measurements of body weight, blood glucose levels, and urinary ketone bodies of SHRs prior to modeling diabetes and at day 25 following administration of streptozotocin (Table 1).

Therefore, all rats exhibited hyperglycosuria and increased urinary ketone body levels. Furthermore, it can be noted that the degree of ketonuria correlated with weight loss from the start of the study until the collection of samples. The greater the weight loss experienced by the animal, the higher the level of urinary ketones was on day 25 of the study.

### 3.2. Hemodynamic Profile of Experimental Groups

Systemic hemodynamic parameters confirmed the expected phenotypic differences between normotensive WKY and hypertensive SHRs, and the metabolic impact of diabetes (Table 2). As anticipated, both 38- and 57-week-old SHR groups exhibited significantly elevated systolic and diastolic blood pressure compared to age-matched WKY controls (*p* ≤ 0.05). Diabetes induction did not significantly alter blood pressure in either WKY+DM or SHR+DM groups relative to their non-diabetic counterparts. However, heart rate was markedly increased in all SHR-based groups (38 w AH, 57 w AH, and AH+DM) compared to controls (*p* ≤ 0.05), with the highest values observed in the comorbid AH+DM group.

These findings confirm the successful establishment of arterial hypertension in SHRs, characterized by significantly elevated systolic and diastolic blood pressure compared to normotensive WKY controls. Induction of insulin-dependent diabetes mellitus did not substantially alter blood pressure levels; however, it was associated with a marked increase in HR in the comorbid AH+DM group. This tachycardia may reflect heightened sympathetic tone or impaired autonomic regulation under conditions of combined metabolic and hemodynamic stress, potentially contributing to adverse cardiac remodeling observed in subsequent histological analyses.

### 3.3. Morphological Characteristics of LV Myocardium in Rats with Different Types of Hypertension, Insulin-Dependent Diabetes, and Their Combination

**Figure 1:**
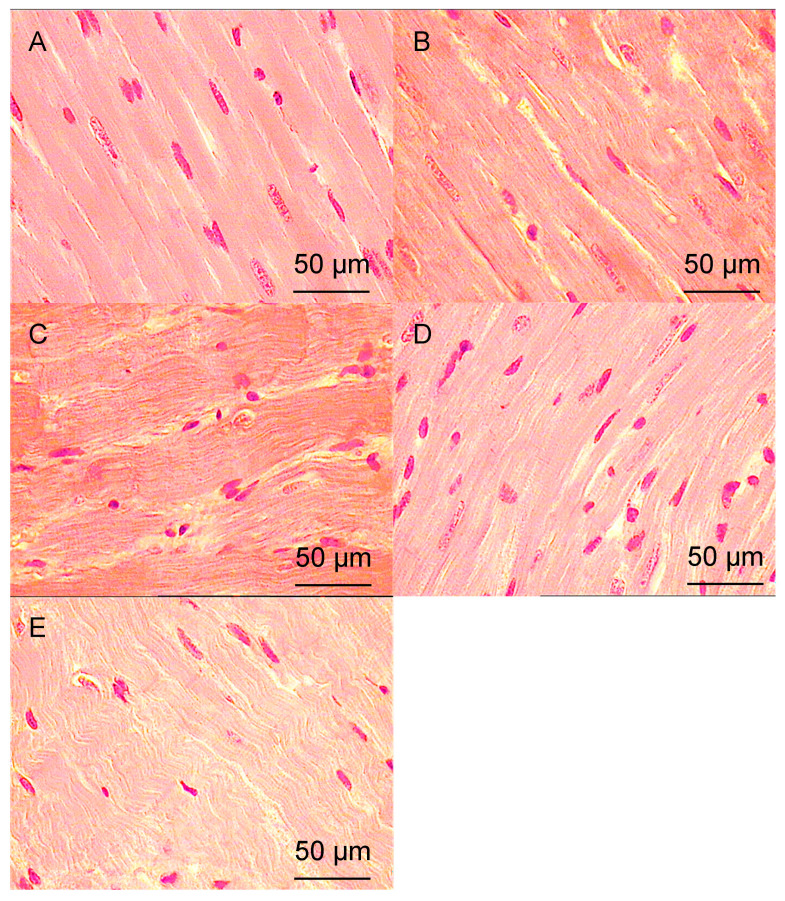
Left ventricular (LV) myocardium of rats. Hematoxylin and eosin staining. Magnification ×400; scale bar = 50 μm (embedded in image). (field width ≈ 270 μm). (**A**)—control; (**B**)—38 weeks of AH; (**C**)—57 weeks of AH; (**D**)—DM; (**E**)—38 weeks of AH + DM.

The following structural features were observed under light microscopy in histological sections of LV myocardium (Figure 1).

In the control group, the myocardial tissue of intact animals did not show any signs of pathological changes. The muscle fibers were clearly defined and mostly lay tightly and in parallel to each other. Tissue, cellular, and intracellular structures were clearly visible. Collagen was present in small amounts, located between myofibrils in thin layers and around microvessels. Areas of destruction were extremely rare and had a small size. Vessels were also rare (Figure 1A).

In a group of 38-week-old hypertensive rats, the CMC hypertrophy in the myocardium was observed, with local areas of spatial orientation disruption and a tendency towards a decrease in nucleus number. The volume of intercellular space and stromal elements decreased slightly compared to the control. Destructive areas were more prevalent. There was a tendency towards an increase in microcirculatory vessel number, but without a significant difference (Figure 1B). Morphometric analysis revealed a significant increase in myofibril volume area and a decrease in the number of nuclei compared to the control, indicating muscle fiber hypertrophy. The decrease in N/C ratio compared to control also supports this finding (Table 3).

In the group of hypertensive animals aged 57 weeks, there was a significant increase in CMC hypertrophy, with a significant increase in the number of sites where their spatial orientation was disrupted, and a tendency to decrease in the number of nuclei per unit area of section persisted. The number of stromal elements increased slightly compared to the group aged 38 weeks, and the area of intercellular space decreased significantly compared to the control group. The number of microvessels continued to increase (Figure 1B). N/C ratio was reduced relative to control, indicating hypertrophy of muscle fibers; however, it increased slightly relative to the group aged 38 weeks (Table 3), consistent with the findings of previous studies on long-term hypertension (>1 year) that did not significantly differ from control groups.

In the DM group, after 30 days, the muscle fibers are predominantly parallel to each other. Hypertrophy of the CMCs is evident, along with thickening of the walls of the microvessels. Areas with impaired spatial orientation are infrequent, and there is a significant increase in the amount of collagen present between the CMCs and around the vessels (Figure 1D). The number of nuclei in the muscle tissue increases slightly compared to the control group, while the number of muscle fibers significantly decreases due to hypertrophy, and the N/C ratio increases relative to the control, indicating CMCs hypertrophy. Additionally, there is a substantial increase in the number of stromal elements compared to the control, suggesting a progressive increase in stromal elements. The volume of intercellular space remains unchanged relative to the control (Table 3).

In a group of animals with AH and DM, CMCs were hypertrophied, and there were areas of disruption in their spatial orientation as well as transverse ruptures in myofibrils (Figure 1E). The number of CMC nuclei was two times lower compared to the control group. The N/C ratio decreased by two times compared to the control, which may suggest partial apoptosis of the CMC mediated by nuclear extrusion. There was a significant increase in the number of stromal elements compared to the control group and a significant decrease in the area of intercellular space. Additionally, there was a significant increase in the number of vessels in the microcirculation bed, with thickened vessel walls compared to the control (Table 3).

Increased collagen deposition was inferred from the expansion of eosinophilic, acellular interstitial spaces on hematoxylin and eosin (H&E)-stained sections, consistent with stromal elements—a methodology validated in our prior studies [22]. Quantitative assessment was performed as “stromal elements, vol.%” in Table 3.

Based on the above-mentioned structural changes, it can be noted that in the DM group, there has been a decrease in the volume fraction of CMCs due to hypotrophy, while the N/C ratio has increased. In the 38-week AH group combined with DM, CMCs have died, as evidenced by a sharp decrease in the number of nuclei and a return to control levels of CMC percentage. Additionally, there has been a two-fold decrease in the N/C ratio compared to the control.

### 3.4. Immunohistochemical Study of HSP90 Expression in the LV Myocardium

**Figure 2:**
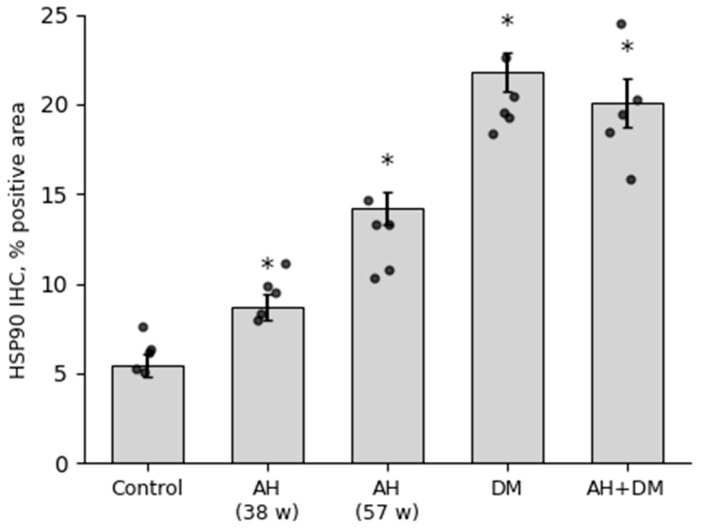
Content of HSP90 in LV CMCs in AH (38- and 57-week-old SHRs), DM (38-week-old WKY rats with insulin-dependent DM), and a combination of AH and DM (38-week-old SHRs with insulin-dependent DM). Data are presented as mean ± SEM (*n* = 5 per group). Statistical significance was assessed by the Mann–Whitney U test with Holm–Bonferroni correction for multiple comparisons (*—*p* ≤ 0.05 vs. Control).

**Figure 3:**
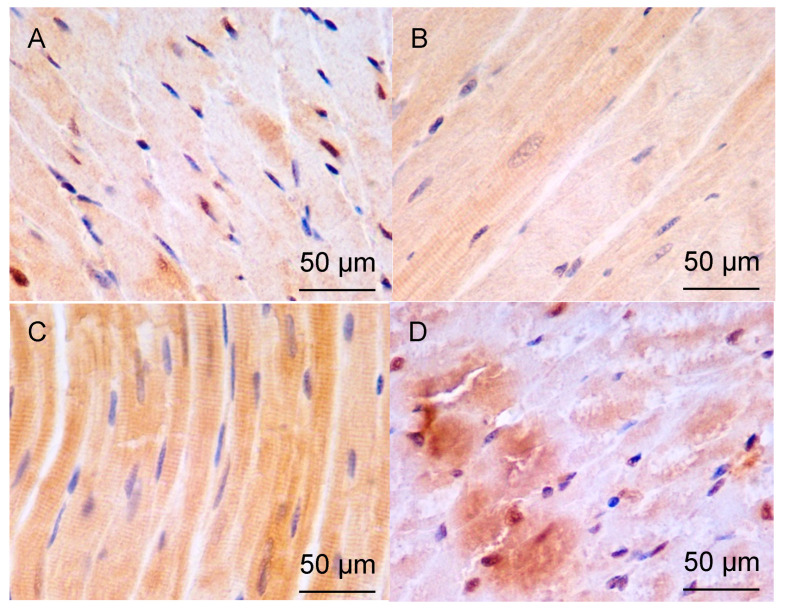
Expression of HSP90 in LV CMCs (middle layer). Immunohistochemical staining, ×400; scale bar = 50 μm (embedded in image). (**A**)—control; (**B**)—AH (57-week-old SHRs); (**C**)—DM (38-week-old WKY rats with insulin-dependent DM); (**D**)—AH+DM (38-week-old SHRs with insulin-dependent DM).

According to the quantitative analysis, a significant increase in HSP90 expression was observed in all the experimental animal groups (Figure 2). The highest level of protein expression was found in the group with isolated insulin-dependent DM, which was more than four times higher than in the control group. In the groups with long-term AH (57 weeks) and in a combination of AH and DM, the protein expression level was approximately the same. However, in the groups with a shorter duration of AH, the protein concentration was slightly higher than in the control group (Figure 2). While HSP90 immunoreactivity in cardiomyocytes dominated the signal, perivascular smooth muscle cells also showed positive staining in diabetic groups (Figure 3C,D), suggesting a potential role in microvascular stress adaptation.

A qualitative analysis of the left ventricular myocardium after an immunohistochemical reaction with HSP90 revealed the following findings (Figure 3). All representative micrographs (Figure 3) depict the mid-myocardial layer, which was systematically analyzed across groups. HSP90 immunoreactivity was predominantly cytoplasmic in cardiomyocytes, exhibiting a diffuse granular pattern throughout the sarcoplasm that aligned with the striated architecture of myofibrils. Nuclear staining was consistently absent. In addition to cardiomyocyte expression, HSP90 showed moderate cytoplasmic positivity in perivascular smooth muscle cells, particularly in diabetic groups (DM and AH+DM), while vascular endothelium remained unstained. No specific mitochondrial, sarcoplasmic reticular, or membrane-associated enrichment was discernible at the resolution of light microscopy (×400).

In the control group, there was positive staining of the cytoplasm in a small number of CMCs. The staining density was highest in the areas of the myocardium near the epicardium, and it decreased towards the endocardial side, eventually disappearing. In the areas surrounding blood vessels, the number of cells showing a positive reaction for HSP90 increased slightly. The staining of CMCs appeared to be predominantly local (Figure 3A).

In the group of 38-week-old hypertensive rats, the density of positively stained CMCs was found to be higher compared to the control group. Localized staining of individual CMC groups was observed, with a relatively higher staining density in the middle layer of the myocardium. The number of positively stained CMCs decreased significantly towards the epicardium and endocardium. No staining was observed around blood vessels.

In the group of hypertensive animals aged 57 weeks, the density of CMCs that had a positive reaction to HSP90 increased. Within a wide area of the myocardium, the staining of just some individual fibers of CMCs was characteristic. Positive-stained CMCs were mainly found in the middle layer of the myocardium and in the layer adjacent to the endocardium (Figure 3B).

In the group of DM, the number of positively stained CMCs increased significantly compared to the group of intact animals. Staining of numerous CMC fibers was observed over a significant length, both in the depth of the myocardium and towards the endocardium and epicardium. Positive staining could be seen around blood vessels, but it was not as intense as in the deep layers of the myocardium (Figure 3C).

For the group of animals with AH and insulin-dependent DM for a period of 38 weeks, intensive staining of a large number of collagen fibers over a significant length in the depth of the myocardium and towards the endocardium was characteristic. There were also fibers with local areas of higher intensity staining. However, the staining was less intense compared to the group with isolated DM (Figure 3D).

### 3.5. Immunohistochemical Study of HSP70 Expression in the LV Myocardium

**Figure 4:**
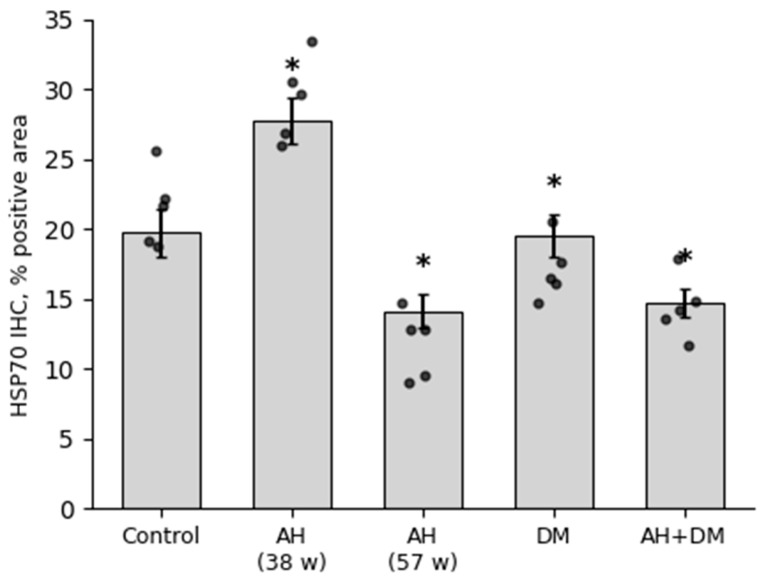
Content of HSP70 in LV CMCs in AH (38- and 57-week-old SHRs), DM (38-week-old WKY rats with insulin-dependent DM), and a combination of AH and DM (38-week-old SHRs with insulin-dependent DM). Data are presented as mean ± SEM (*n* = 5 per group). Statistical significance was assessed by the Mann–Whitney U test with Holm–Bonferroni correction for multiple comparisons (*—*p* ≤ 0.05 vs. Control).

According to the results of this study, obtained on the basis of quantitative analysis, there was a statistically significant increase in the expression of the HSP70 protein in the group of animals with shorter periods of AH (38 weeks) (Figure 4). In the group with isolated insulin-dependent DM, the protein expression remained at the control levels. In the animals with longer periods of AH (57 weeks) and a combination of AH and DM, the expression of HSP70 decreased relatively to that of the control group (Figure 4).

**Figure 5:**
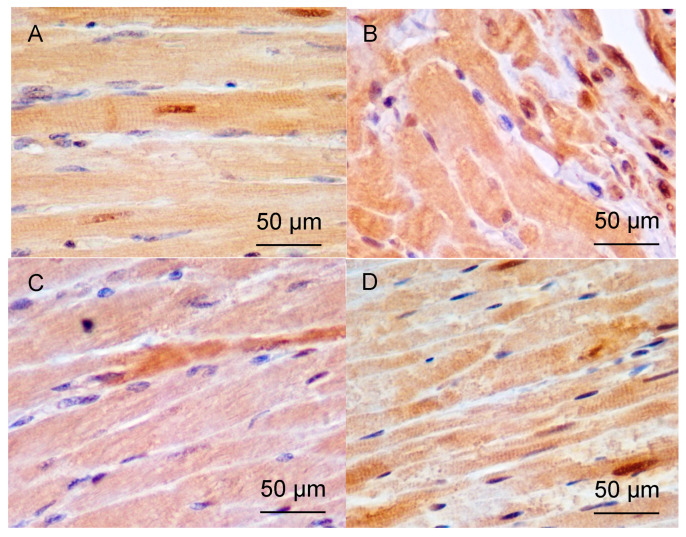
Expression of HSP70 in LV CMCs (middle layer). Immunohistochemical staining, ×400; scale bar = 50 μm (embedded in image). (**A**)—control; (**B**)—AH (38-week-old SHRs); (**C**)—DM (38-week-old WKY rats with insulin-dependent DM); (**D**)—AH+DM (38-week-old SHRs with insulin-dependent DM).

A qualitative analysis of sections of the LV myocardium after an immunohistochemical reaction to HSP70 revealed the following features (Figure 5). All representative micrographs (Figure 5) depict the mid-myocardial layer, which was systematically analyzed across groups. HSP70 immunoreactivity was exclusively cytoplasmic in cardiomyocytes, with diffuse granular staining distributed throughout the sarcoplasm and absent from nuclei. The signal co-localized with the characteristic cross-striated pattern of myofibrils, confirming its localization within contractile cardiomyocyte cytoplasm. No enrichment at the sarcolemma, intercalated discs, or perinuclear regions was observed. In diabetic groups (DM and AH+DM), weak to moderate HSP70 staining was also noted in perivascular smooth muscle cells, while endothelial cells remained negative.

In the control group, positive staining is primarily concentrated in the depth of the myocardium and towards the epicardial direction. Staining did not extend towards the endocardial region. Within the deep layers of the myocardium, the staining appeared continuous, while in the epicardial orientation, individual CMCs or clusters of CMCs exhibited staining (Figure 5A).

At 38 weeks of AH, the positive immunohistochemical reaction was mainly concentrated in the myocardium and was much more intense than in the control group. The intensity of staining in the epicardial direction decreased compared to the internal layers, but individual stained CMCs and their groups remained. The intensity of staining also decreased in the endocardial direction and eventually disappeared. Staining around blood vessels increased, including in smooth muscle cells of the vascular wall (Figure 5B).

At 57 weeks of AH positive reaction was seen with separate-colored fibers of CMCs in the depth of the myocardium. Towards the epicardium, the staining intensity decreased, and individual groups of CMCs became stained towards the endocardium. Near the epicardium, the staining gradually decreased until it disappeared completely. The staining around blood vessels was less intense than in the AH group at 38 weeks.

In the case of DM, intense staining was detected around blood vessels, similar to that of the 38-week AH group. The staining was mainly concentrated in the deep layers of the myocardium, characterized by high intensity in individual CMC fibers. Intense staining of CMCs could be seen in the center and towards the epicardium. Towards the endocardium, the intensity of the staining decreased and became more diffuse (Figure 5C).

In a combination of AH and DM, the staining of medium intensity was typical, it was continuous and mainly located in the depth of the myocardium. Separate groups of CMCs with higher intensity could be seen in the direction of the endocardium. Towards the epicardium, the staining became less intense, and marked staining could be found around blood vessels. This staining pattern was similar to that in the group of animals with isolated DM (Figure 5D).

### 3.6. RT-PCR Analysis of mRNA HSP70 and HSP90 Expression in the LV Myocardium

**Figure 6:**
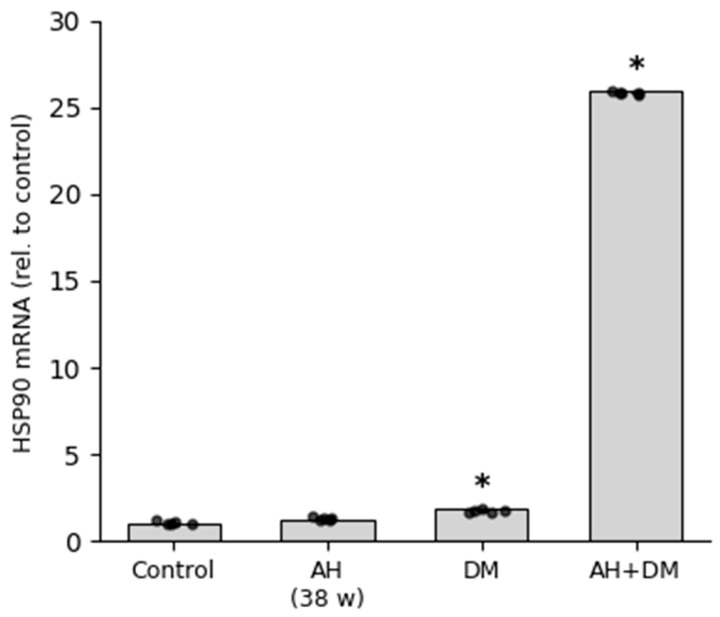
HSP90 mRNA expression in rat CMCs. Relative mRNA levels were quantified by RT-qPCR and normalized to the control group (set as 1.0) using the 2^−^ΔΔCt method. Data are presented as mean ± SEM (*n* = 5 per group). Groups: Control; AH—arterial hypertension (38 weeks); DM—diabetes mellitus (30 days); AH+DM—comorbidity of arterial hypertension and diabetes mellitus. *—*p* ≤ 0.05 vs. Control.

**Figure 7:**
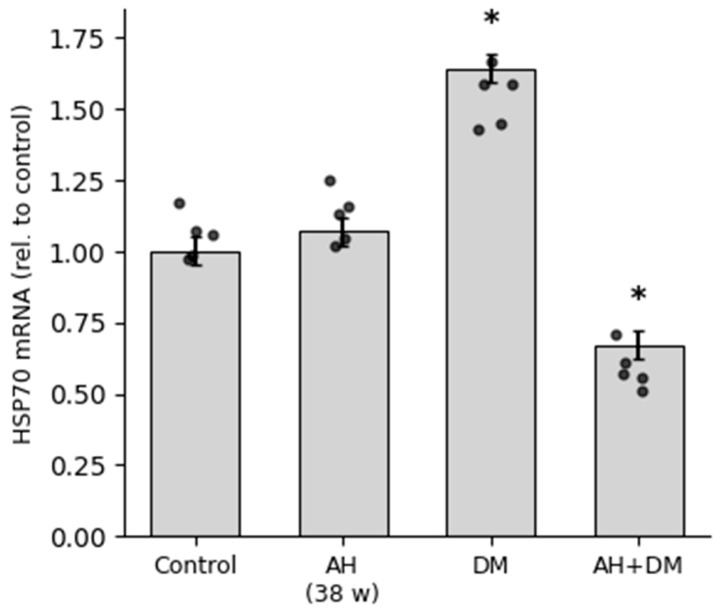
HSP70 mRNA expression in rat CMCs. Relative mRNA levels were quantified by RT-qPCR and normalized to the control group (set as 1.0) using the 2^−^ΔΔCt method. Data are presented as mean ± SEM (*n* = 5 per group). Groups: Control; AH—arterial hypertension (38 weeks); DM—diabetes mellitus (30 days); AH+DM—comorbidity of arterial hypertension and diabetes mellitus. *—*p* ≤ 0.05 vs. Control.

HSP90 and HSP70 mRNA expression was differentially regulated in rat cardiomyocytes in response to arterial hypertension (AH), diabetes mellitus (DM), and their comorbidity. RT-qPCR analysis revealed a moderate increase in HSP90 mRNA levels in the AH group and a more pronounced upregulation in the DM group. Strikingly, the comorbid AH+DM condition induced an extreme and highly significant elevation of HSP90 mRNA expression, suggesting a synergistic stress response at the transcriptional level (Figure 6). In contrast, HSP70 mRNA expression showed an initial upregulation in the DM group but was significantly downregulated under comorbid conditions. Expression in the AH group remained comparable to control levels (Figure 7). This pattern reflects reduced HSP70 expression in the context of combined hypertensive and diabetic stress.

### 3.7. Comparative Description of RT-qPCR and IHC Data

**Figure 8:**
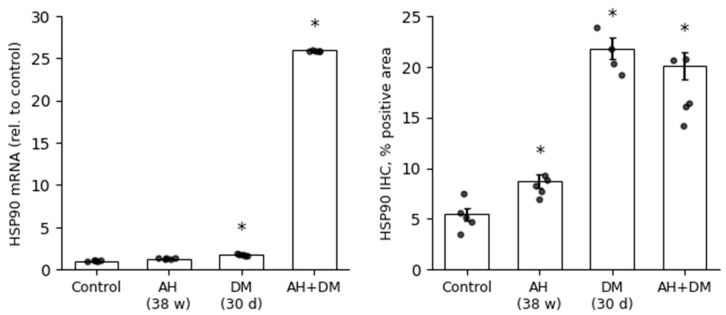
Concordant upregulation of HSP90 at mRNA and protein levels in rat cardiomyocytes under comorbid arterial hypertension and diabetes mellitus. Protein expression was quantified by IHC as the percentage of positively stained area. mRNA expression was determined by RT-qPCR and normalized to the control group (set as 1.0) using the 2^−^ΔΔCt method. Data are presented as mean ± SEM (*n* = 5 per group). Statistical significance was assessed by the Mann–Whitney U test with Holm–Bonferroni correction for multiple comparisons (*—*p* ≤ 0.05 vs. Control). Groups: Control; AH—arterial hypertension (38 weeks); DM—diabetes mellitus (30 days); AH+DM—comorbidity of arterial hypertension and diabetes mellitus.

**Figure 9:**
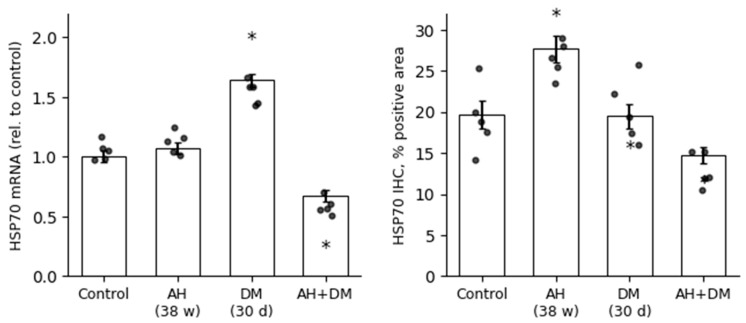
Divergent regulation of HSP70 at mRNA and protein levels in rat cardiomyocytes under comorbid stress. Protein expression was assessed by IHC as the percentage of positively stained area. mRNA expression was measured by RT-qPCR and normalized to the control group (set as 1.0) using the 2^−^ΔΔCt method. Data are presented as mean ± SEM (*n* = 5 per group). Statistical significance was assessed by the Mann–Whitney U test with Holm–Bonferroni correction for multiple comparisons (*—*p* ≤ 0.05 vs. Control). Groups: Control; AH—arterial hypertension (38 weeks); DM—diabetes mellitus (30 days); AH+DM—comorbidity of arterial hypertension and diabetes mellitus.

A comparative analysis of mRNA and IHC data was performed to assess concordance between transcriptional activity and protein-level responses (Figure 8 and Figure 9). While IHC-derived staining area does not equate to absolute protein concentration, it provides a reliable index of relative chaperone expression and subcellular localization in fixed tissue, particularly when combined with mRNA data to assess transcriptional vs. post-transcriptional regulation. It should be noted that IHC quantification reflects the relative distribution and semi-quantitative abundance of HSP90 and HSP70, expressed as the percentage of positively stained cytoplasmic area in cardiomyocytes—a stereological approach widely used for comparative tissue analysis. This metric does not equate to absolute protein concentration but provides a robust index of chaperone expression in fixed tissue when standardized across groups.

In stark contrast, HSP70 exhibited a profound disconnect between its mRNA and protein levels, particularly under comorbid conditions (Figure 9). While RT-qPCR showed a moderate upregulation of HSP70 mRNA in the DM group (1.64-fold), this trend was completely reversed in the AH+DM group, where mRNA levels were significantly downregulated (0.67-fold vs. control). However, the IHC data revealed an even more pronounced loss of HSP70 protein, with the positive area in the AH+DM group dropping to 14.71% ± 0.96% compared to 19.70% ± 1.73% in the control. Notably, in the AH (38 w) group, a transient increase in HSP70 protein (27.68% ± 1.63%) was not accompanied by a corresponding rise in mRNA (1.07-fold), suggesting a potential role for post-transcriptional regulatory mechanisms in the early adaptive response to hypertension.

Collectively, these findings indicate that while HSP90 expression is primarily driven by transcriptional activation, the regulation of HSP70 is more complex, involving both transcriptional and post-transcriptional control mechanisms, which appear to be overwhelmed or dysregulated in the setting of AH and DM comorbidity.

## 4. Discussion

As a result of our experiments, we found an increase in the expression of the heat shock protein HSP90 in all the pathology groups. The most pronounced change was observed in the group with isolated DM. Conversely, the expression of heat shock protein HSP70 only increased in the group with AH of a shorter duration and remained at the control level in the group with isolated DM.

The increase in HSP90 protein expression observed in all pathological groups may reflect its involvement in stress-responsive signaling pathways activated during cardiac remodeling. Previous studies have shown that HSP90 modulates the synthesis and release of IL-6 via exosomes, which in turn activates signal transducer and transcription activator 3 (STAT3) in cardiac fibroblasts, promoting extracellular matrix expansion under hypertrophic conditions [31]. Furthermore, pharmacological inhibition of HSP90 with 17-DMAG was shown to attenuate fibroblast activation and structural changes in the vascular adventitia of hypertensive mice [32], suggesting a broader role for HSP90 in tissue remodeling responses to hemodynamic and metabolic stress. In our model, the pronounced upregulation of HSP90—particularly under comorbid conditions—may therefore represent an adaptive chaperone response to sustained proteotoxic and inflammatory stimuli, rather than a direct driver of structural pathology.

An increase in the level of HSP70 expression in short-term AH correlates with the data demonstrating that acute hypertension induces the rapid expression of HSP70 mRNA, followed by an increase in HSP70 protein levels in the rat aorta [33]. Another group of scientists also concluded that essential hypertension contributes to an increase in circulating HSP70 levels and HSP70 expression at the mRNA level, and they found a relationship between circulating HSP70 levels and inflammatory markers in blood plasma. However, HSP70 is an important endogenous antigen in essential hypertension, and inflammation resulting from the activation of innate and adaptive immune factors is involved in the development and maintenance of high blood pressure. Experimental induction of tolerance to HSP70 leads to the production of an IL-10-regulated response by T cells that prevents inflammation in patients with essential hypertension [34].

As regards the decrease in intracellular expression of HSP70 in long-term hypertension, this may be related to the translocation of the protein from cardiomyocytes into the extracellular space, where it can act as a damage-associated molecular pattern (DAMP) and contribute to systemic inflammatory responses associated with chronic hypertension. This interpretation is supported by previous evidence showing that sustained overexpression of HSP70 in cardiomyocytes does not prevent the development of structural and functional alterations in chronic cardiac pathology [35]. Thus, while HSP70 induction may support cellular adaptation during acute stress, its intracellular availability appears to be diminished under prolonged hemodynamic and metabolic strain, potentially limiting its role in maintaining proteostasis in chronic disease settings.

The observed discordance between HSP70 mRNA and immunoreactivity—particularly the marked protein loss despite modest mRNA changes—suggests that post-transcriptional mechanisms (e.g., microRNA-mediated degradation, altered protein turnover) may dominate HSP70 regulation under comorbid stress. Although not absolute, IHC-based assessment more directly captures regional changes in myocardial HSP70 protein compared with transcript-level measurements.

The comparison of SHR values at the age of 38 weeks (about 9 months) and 57 weeks (about 13–14 months) is associated with the influence of the age factor, regardless of the severity of hypertension. Indeed, it is well documented in the literature that the expression and functional activity of HSP70 and HSP90 decrease with age in the heart and other tissues due to attenuation of HSF1 transcriptional activity, accumulation of damaged proteins overloading the chaperone system, and epigenetic suppression of HSP gene promoters (for example, through hypermethylation). In our study, HSP70 did not show a significant increase even in the SHR+DM group, which may reflect not only the specifics of comorbid pathology, but also the age-associated depletion of the stress response in 38-week-old animals. This is consistent with data showing that in rats older than 6 months, the induction of HSP70 in response to hypoxia, ischemia, or hyperglycemia becomes less pronounced or delayed compared to younger individuals [36,37].

It is important to note that 57-week-old SHRs are approaching senescence for laboratory rodents, an age at which structural and functional signs of cardiac aging—such as diastolic dysfunction and mitochondrial insufficiency—become evident. Under these conditions, even the basal expression of heat shock proteins may be altered, complicating direct comparisons with 38-week-old animals, which represent a stage of mature but still compensated hypertension.

Thus, differences in HSP70 expression observed across SHR age groups likely reflect a combination of factors, including the duration of hypertensive exposure, the stage of metabolic dysregulation in the presence of diabetes, and age-associated modulation of cellular stress-response pathways. Future studies should include age- and sex-matched control groups and assess HSF1 activity as well as post-translational modifications of HSPs to better disentangle the respective contributions of pathology and aging.

As concerns the expression of the HSP70 protein in DM at the level of the control group, different studies have reported discrepancies. Both a decrease [36] and an increase [36] in its levels have been reported, as well as no change between the groups [38]. However, in patients with DM and concomitant obesity, the levels of intracellular HSP70 in skeletal muscle and myocardium are reduced. This may be associated with insulin resistance [39]. In primates, increasing the level of intracellular HSP70 through modeling diabetes was shown to be an effective method for restoring glucose tolerance [40]. Higher expression of HSP70 in skeletal muscles also protects against the development of insulin resistance during aging [41]. Therefore, direct or indirect restoration of HSP70 may have a positive effect on insulin signaling.

The divergent regulation of HSP70 and HSP90 observed in our study—particularly the pronounced upregulation of HSP90 and concomitant suppression of HSP70 in the AH+DM comorbidity group—likely reflects distinct upstream signaling mechanisms activated under combined metabolic and hemodynamic stress.

HSP90 expression is tightly controlled by multiple stress-responsive pathways, including NF-κB, STAT3, and the unfolded protein response (UPR). In the setting of diabetes, chronic hyperglycemia and accumulation of advanced glycation end products (AGEs) activate the RAGE–NF-κB axis, which has been shown to enhance HSP90 transcription and stabilize client proteins involved in inflammatory and remodeling processes [19,30]. Moreover, IRE1α, a key sensor of endoplasmic reticulum stress, forms a stable complex with HSP90 to promote its own activation and downstream splicing of XBP1, a transcription factor that further upregulates chaperone and stress-adaptive genes [17,18]. This mechanism may contribute to the elevated HSP90 levels observed in our DM and AH+DM groups. Additionally, angiotensin II signaling—chronically activated in SHRs—stimulates NADPH oxidase–dependent ROS production, which in turn activates STAT3 via phosphorylation. Activated STAT3 not only modulates extracellular matrix dynamics in cardiac fibroblasts [30] but also directly enhances HSP90 transcription, creating a feed-forward loop that exacerbates myocardial structural remodeling under comorbid conditions.

In contrast, HSP70 expression is primarily governed by heat shock factor 1 (HSF1), whose activity is highly sensitive to redox balance, energy status, and post-translational modifications. Under acute stress (e.g., short-term AH), HSF1 trimerizes, translocates to the nucleus, and induces HSP70 transcription—consistent with the transient increase we observed in the 38-week SHR group. However, in chronic hyperglycemia and aging, HSF1 function becomes impaired due to oxidative inactivation, sequestration by misfolded proteins, or inhibitory phosphorylation by kinases such as GSK-3β and ERK1/2 [42,43]. Furthermore, persistent ER stress in diabetes may divert HSF1 co-factors toward UPR-mediated transcription, further dampening the canonical heat shock response. This age- and pathology-dependent attenuation of HSF1 activity likely underlies the blunted HSP70 induction in the DM and AH+DM groups, despite elevated cellular stress.

Notably, the discrepancy between HSP70 mRNA and protein levels in the AH+DM group (Figure 8) suggests additional post-transcriptional regulation. MicroRNAs such as miR-1 and miR-206, which are upregulated in diabetic cardiomyopathy, can directly target HSP70 mRNA for degradation [44]. Alternatively, increased protein turnover via chaperone-mediated autophagy or ubiquitin–proteasome degradation—both accelerated under oxidative stress—may reduce HSP70 protein stability without affecting transcript abundance.

Taken together, these data indicate that HSP90 is upregulated through inflammatory and stress-responsive signaling pathways—including NF-κB, STAT3, and the UPR—whereas HSP70 induction is blunted due to age- and diabetes-related impairment of HSF1 activity and enhanced post-transcriptional regulation [36,37]. This imbalance may reflect a shift in cellular stress-response regulation under combined hypertensive and diabetic conditions and may be associated with molecular changes linked to myocardial structural remodeling.

This study has several limitations. First, we did not assess in vivo cardiac function or systemic biomarkers (e.g., troponin, insulin), as our ethical protocol was restricted to terminal histological and molecular endpoints. Second, extracellular matrix expansion was evaluated on H&E-stained sections rather than collagen-specific stains; however, stromal volume fraction remains a validated morphometric indicator in our experimental model. Third, cellular compartmentalization of protein expression (e.g., cardiomyocyte vs. vascular) was based on qualitative immunohistochemical assessment. Future studies will incorporate functional imaging, specialized matrix staining, and laser capture microdissection to further resolve cell-type-specific stress responses in the myocardium. Accordingly, our findings should be interpreted as molecular/histological correlations of stress response rather than evidence of preserved cardiac function.

## 5. Conclusions

Based on the results of our study, we can conclude that HSP90 expression is upregulated in all pathological conditions—arterial hypertension, insulin-dependent diabetes mellitus, and their comorbidity—likely reflecting its involvement in stress-responsive signaling and extracellular matrix remodeling. The magnitude of HSP90 induction appears to correlate with the severity of the combined metabolic and hemodynamic insult, particularly in the AH+DM group. In contrast, HSP70 expression increases only during the early phase of hypertension, suggesting a transient adaptive response, while remaining at baseline levels in diabetes and declining under comorbid stress. This pattern is consistent with altered regulation of HSP70-associated protein quality control pathways in the myocardium and may be associated with molecular features linked to structural remodeling in chronic cardiometabolic disease.

## Figures and Tables

**Table 1:** Body weight, urine ketone body levels, and urine glucose levels in SHRs with streptozotocin-induced insulin-dependent DM on day 25 after induction of DM (*n* = 5). Data correspond exclusively to Group 5 (AH+DM).

SHR+DM	1	2	3	4	5
Body weight before modeling DM, g	315	331	292	314	354
Body weight after 25 days of administration of streptozotocin, g	254	231	216	218	306
Glucose in urine (mmol/L)	>112	>112	>112	>112	>112
Ketone bodies in urine (mmol/L)	0.5	4	1.5	1.5	0.5

**Table 2:** Values of systolic and diastolic blood pressure (BPsyst, BPdiast) and heart rate (HR) in control, after 38 weeks of AH, 57 weeks of AH, DM, and a combination of 38-week AH and DM. Data are presented as mean ± SEM (*n* = 5 per group). Statistical significance was assessed by the Mann–Whitney U test with Holm–Bonferroni correction for multiple comparisons (*p* ≤ 0.05 vs. Control). Asterisks (*) in the table denote significant differences relative to the control group.

	Control	38 Weeks of AH	57 Weeks of AH	DM	38 Weeks of AH + DM
BPsyst, mmHg	116.4 ± 2.1	194.0 ± 10.05 *	196.33 ± 4.37 *	115.3 ± 2.2	189.0 ± 8.4 *
BPdiast, mmHg	82.6 ± 3.8	132.5 ± 8.1 *	138.6 ± 2.1 *	78.7 ± 2.9	130.2 ± 6.2 *
HR, bpm	250.3 ± 6.1	334.7 ± 5.4 *	340.2 ± 4.1 *	246.1 ± 5.7	350.2 ± 7.4 *

**Table 3:** Morphometric analysis of histological sections of left ventricular (LV) myocardium in control, after 38 weeks of AH, 57 weeks of AH, DM, and a combination of 38-week AH and DM. Data are presented as mean ± SEM (*n* = 5 per group). Statistical significance was assessed by the Mann–Whitney U test with Holm–Bonferroni correction for multiple comparisons (*p* ≤ 0.05 vs. Control). Asterisks (*) in the table denote significant differences relative to the control group.

Value	Control	AH 38 Weeks	AH 57 Weeks	DM 30 Days	AH 38 Weeks + DM
CMC, vol.%	83.81 ± 0.7	87.2 ± 0.87 *	89.06 ± 1.01 *	79.00 ± 0.82 *	83.50 ± 0.93
Nuclei, vol.%	5.19 ± 0.19	4.3 ± 0.24 *	4.5 ± 0.24 *	5.89 ± 0.36 *	2.85 ± 0.22 *
Nuclear–cytoplasmic ratio (N/C)	0.06 ± 0.003	0.04 ± 0.008	0.05 ± 0.001	0.07 ± 0.001	0.03 ± 0.004
Intercellular space, vol. %	5.61 ± 0.41	4.28 ± 0.44 *	2.66 ± 0.44 *	5.81 ± 0.50	2.07 ± 0.30 *
Stromal volume fraction, vol.%	4.81 ± 0.24	3.44 ± 0.27 *	2.81 ± 0.44 *	7.18 ± 0.47 *	9.43 ± 0.52 *
Vessels, vol. %	0.43 ± 0.26	0.52 ± 0.34	0.62 ± 0.24	1.61 ± 0.62 *	1.84 ± 0.74 *
Destruction sites, vol. %	0.09 ± 0.07	0.22 ± 0.17 *	0.30 ± 0.24 *	0.44 ± 0.08 *	0.28 ± 0.15 *

## Data Availability

Data are contained within the article.
